# Evaluating p-tau217 and p-tau231 as Biomarkers for Early Diagnosis and Differentiation of Alzheimer’s Disease: A Narrative Review

**DOI:** 10.3390/biomedicines12040786

**Published:** 2024-04-03

**Authors:** Dorian Julian Jarek, Hubert Mizerka, Jarosław Nuszkiewicz, Karolina Szewczyk-Golec

**Affiliations:** 1 Student Research Club of Medical Biology and Biochemistry, Department of Medical Biology and Biochemistry, Faculty of Medicine, Ludwik Rydygier Collegium Medicum in Bydgoszcz, Nicolaus Copernicus University in Toruń, 85-092 Bydgoszcz, Poland; 310894@stud.umk.pl; 2 Department of Medical Biology and Biochemistry, Faculty of Medicine, Ludwik Rydygier Collegium Medicum in Bydgoszcz, Nicolaus Copernicus University in Toruń, 85-092 Bydgoszcz, Poland; jnuszkiewicz@cm.umk.pl

**Keywords:** Alzheimer’s disease, amyloid plaques, biomarker, diagnosis, neurodegenerative diseases, neurofibrillary tangles, p-tau, p-tau217, p-tau231, tau proteins

## Abstract

The escalating prevalence of Alzheimer’s disease (AD) highlights the urgent need to develop reliable biomarkers for early diagnosis and intervention. AD is characterized by the pathological accumulation of amyloid-beta plaques and tau neurofibrillary tangles. Phosphorylated tau (p-tau) proteins, particularly p-tau217 and p-tau231, have been identified as promising biomarker candidates to differentiate the disease progression from preclinical stages. This narrative review is devoted to a critical evaluation of the diagnostic accuracy, sensitivity, and specificity of p-tau217 and p-tau231 levels in the detection of AD, measured in plasma, serum, and cerebrospinal fluid, compared to established biomarkers. Additionally, the efficacy of these markers in distinguishing AD from other neurodegenerative disorders is examined. The significant advances offered by p-tau217 and p-tau231 in AD diagnostics are highlighted, demonstrating their unique utility in early detection and differential diagnosis. This comprehensive analysis not only confirms the excellent diagnostic capabilities of these markers, but also deepens the understanding of the molecular dynamics of AD, contributing to the broader scientific discourse on neurodegenerative diseases. This review is aimed to provide key information for researchers and clinicians across disciplines, filling interdisciplinary gaps and highlighting the role of p-tau proteins in revolutionizing AD research and clinical practice.

## 1. Introduction

Since the first diagnosis of Alzheimer’s disease (AD), published by Alois Alzheimer [1,2] on 3 November 1906, more than a century has passed. AD is the most common cause of cognitive impairment among older adults. It affects approximately 3–4% of the population aged 60 years and older [3]. A global status report on the public health response to dementia issued by the World Health Organization (WHO) states that, in 2019, 55.2 million people worldwide were living with dementia [4]. However, a recent study estimated that 22% of individuals worldwide aged 50 years or older have AD [5]. When including patients in the earlier stages of the disease, this estimate rises to a total of 416 million people worldwide, with figures ranging between 327 and 525 million. According to the latest systematic literature review, numerous cases of AD might not be captured in studies including only individuals with a formal diagnosis of AD dementia [6]. The incidence of AD has been increasing in recent years, and it is estimated that this trend will continue [3]. The prevalence of dementia in Europe is expected to double by 2050 [7].

Currently, three main stages of AD are recognized as preclinical AD, AD with mild cognitive impairment (MCI), and dementia [3,8,9]. The earliest phase, preclinical AD, is clinically asymptomatic, but in this phase of the disease, it is possible to identify diagnostic biomarkers of the disease [10,11]. MCI is defined by the initial stage symptoms, which typically include the impairment of short-term memory, followed by a decline in additional cognitive domains [12,13]. The dementia stage is characterized by cognitive impairment severe enough to impact the daily life of a person with AD [7,14].

AD is related to age, and a greater risk of developing AD is observed in women [15], which is probably caused by menopause and the associated disruption of the functioning of estrogen-regulated systems [16,17]. Several genes are significantly associated with AD. Mutations in the amyloid protein precursor (*APP*) and the presenilin-1/2 (*PSEN1/2*) genes account for less than 5% of all AD cases [15,18]. Allelic variation of the apolipoprotein E (*APOE*) gene, specifically the type ε4 allele, is a major risk factor in sporadic AD [15,19]. This allele is 2–3 times more frequent in individuals with AD compared to normal control cases [15]. Modifiable risk factors for AD have also been identified [20,21,22]. Significant risk factors for developing AD include hyperhomocysteinemia and depression [23,24]. Other risk-enhancing factors include pre-existing diseases such as frailty, carotid atherosclerosis, hypertension, low diastolic blood pressure, and type 2 diabetes mellitus in the Asian population, as well as lifestyle factors like low education, increased body mass index (BMI) in mid-life, and low BMI in later life [22,25,26].

The molecular mechanism of AD is determined by the accumulation of both β-amyloid (Aβ) in the form of extracellular neuritic plaques and hyperphosphorylated tau protein in the form of intracellular neurofibrillary tangles (NFTs) [27,28]. Aβ plaques are produced by sequential cleavage of APP by β-secretase (BACE-1) and γ-secretase within cells [29,30]. This process results in the formation of Aβ monomers, which are secreted outside the cell into the intercellular space [31]. Then, they aggregate to form Aβ oligomers, protofibrils, and fibrils [32,33]. These aggregates lead to the hyperphosphorylation of tau, a decrease in cerebral capillary blood flow, and the impairment of the function of synapses [27,28]. In vitro studies have underscored the ability of tau proteins to promote the assembly and stabilization of axonal microtubules (MTs), a process vital for proper neuronal function and axonal transport [28]. Under normal conditions, the interaction between tau and MTs is precisely regulated by phosphorylation, which adjusts tau’s binding affinity to MTs and its neuronal distribution. However, in AD and related tauopathies, tau undergoes aberrant hyperphosphorylation, significantly diminishing its affinity for MTs [28,34]. Consequently, hyperphosphorylated tau accumulates in the cytosol, leading to aggregation and fibrillization. This process results in the formation of NFTs, insoluble aggregates that are characteristic of AD pathology [28,34]. Such pathological aggregation disrupts MT stability and induces neuronal dysfunction by redistributing tau from axonal to somatodendritic compartments. This redistribution hinders essential neuronal processes, including glutamate receptor trafficking and synaptic anchoring, exacerbating synaptic dysfunction and contributing to the cognitive decline seen in AD [28]. Additionally, recent research indicates that the spread of tau aggregates might occur through a prion-like mechanism in which misfolded tau proteins catalyze the misfolding of normal tau, promoting the progression of pathology across neuronal networks [35,36]. The described mechanisms are associated with the clinical and neuropathological stages of AD, underscoring the critical role of tau pathology in the disease evolution and its potential as a therapeutic target. Moreover, NFTs can spread trans-synaptically to anatomically connected brain regions, causing further formation of NFTs in remote parts of the brain, spreading from the entorhinal cortex to the neocortex [28]. The process of NFT production appears to correlate with Aβ deposition [37]. The progression of hyperphosphorylated tau to other parts of the brain occurs only in the presence of Aβ, suggesting a mechanistic link [28]. However, the presence of Aβ, unlike the stage of tau pathology, does not correlate with the progression of cognitive impairment, indicating the significant role of tau hyperphosphorylation in inducing AD symptoms [28].

Given the critical need for advancements in the early detection and differentiation of AD amid its growing global prevalence, this narrative review sets forth to critically evaluate the diagnostic utility of phosphorylated-tau (p-tau) proteins, specifically p-tau Thr217 (p-tau217) and p-tau Thr231 (p-tau231), as potential biomarkers for AD. This evaluation synthesizes findings from a broad spectrum of studies to elucidate the sensitivity, specificity, and overall diagnostic accuracy of these biomarkers across the various stages of AD, including the preclinical and prodromal phases. Furthermore, this review aims to contrast the effectiveness of p-tau217 and p-tau231 against other established biomarkers for AD, examine their potential in distinguishing AD from other neurodegenerative disorders, and explore the practical implications of their application in clinical settings. By delving into these areas, the review endeavors to fill existing gaps in interdisciplinary research and underscore the transformative role of p-tau proteins in revolutionizing AD research and clinical practice.

## 2. Current Diagnosis of AD

The diagnosis of AD is based on the 2011 recommendations of the National Institute on Aging–Alzheimer’s Association (NIA-AA) workgroups on diagnostic guidelines for Alzheimer’s disease [38]. Unlike the recommendations for diagnosing MCI due to AD and AD dementia stage, recommendations for preclinical AD are not intended for clinical diagnosis and are based only on the presence of biomarkers, due to the asymptomatic course of this phase of AD. The diagnosis of MCI due to AD requires the establishment of clinical and cognitive criteria. These criteria include a change in cognition reported by a patient, an informant, or a clinician; objective evidence of impairment in one or more cognitive domains—such as memory, executive function, attention, language, and visuospatial skills—obtained through testing; maintenance of the patient’s independence in functional abilities; and no dementia in a patient [39]. Additionally, it is necessary to assess the etiology of MCI to ensure it is consistent with the AD pathophysiological process [39]. The diagnosis of AD dementia is based on symptoms of cognitive decline that develops over time and interferes with the patient’s usual activities. The cognitive or behavioral impairment must involve at least two of the previously mentioned domains [40,41]. It is crucial to exclude other causes of dementia, such as delirium or major psychiatric disorders [40,42]. The cognitive impairment should be detected by history-taking from the patient and other informants, as well as objective forms of cognitive assessments by neurophysiological testing [40]. Additionally to the mentioned-above core criteria for the diagnosis of MCI due to AD and AD dementia, in the 2011 recommendations from the NIA-AA, biomarkers have been established to support the diagnosis of both stages [38,39,40]. Aβ deposition is the first biomarker, examined by the presence of the Aβ42 variant of Aβ in the cerebrospinal fluid (CSF) or by detection during positron emission tomography (PET) amyloid imaging [40,43]. The second set of biomarkers is related to neuronal injury and includes the presence of phosphorylated-tau in the CSF, volumetric measurements or visual assessments of hippocampal volume or medial temporal lobe atrophy, the rate of brain atrophy, fluorodeoxyglucose-18 (FDG)-PET imaging, and single-photon emission computed tomography (SPECT) perfusion imaging. Additionally, there are less validated methods such as functional magnetic resonance imaging (fMRI) activation studies, resting blood oxygen level-dependent (BOLD) functional connectivity, MRI perfusion, magnetic resonance spectroscopy, diffusion tensor imaging, and voxel-based and multivariate measures [39,40,43]. The biomarkers of the third type include inflammatory biomarkers (cytokines), oxidative stress biomarkers (isoprostanes), and other markers of synaptic damage and neurodegeneration such as cell death [39,40].

## 3. New ‘Candidates’ for AD Biomarkers

For diagnostic purposes, the currently used p-tau biomarker includes p-tau phosphorylated at Thr181 (p-tau181) [44,45,46]. Recently, new ‘candidate’ p-tau biomarkers have been proposed, namely p-tau217 and p-tau231 [44,47,48,49]. Studies have demonstrated that pre-screening with blood testing could significantly reduce the need for more invasive testing in AD, and that plasma p-tau may offer greater diagnostic power than plasma amyloid measures [47]. It has been shown that p-tau181 and p-tau217 can differentiate between amyloid-PET or tau-PET positive cases and amyloid-PET or tau-PET negative cases. Furthermore, the p-tau level allows us to distinguish patients with AD dementia from those with frontotemporal lobar degeneration [47]. Recent studies have revealed increased levels of p-tau217 in the plasma of individuals in the preclinical and early clinical stages of AD compared to a control group of healthy individuals [49]. This increase is also associated with a higher risk of progressing to AD dementia, a faster rate of cognitive decline, and thinning of the temporal cortex and hippocampus. The authors suggest the possibility of monitoring treatment responses over time by comparing p-tau217 levels in non-demented, amyloid-positive individuals and those without such pathology, as well as in non-demented individuals who later develop AD dementia compared to those who remain non-demented during follow-up, highlighting a greater increase in AD-related individuals [49]. They advocate for the use of p-tau217 in monitoring the disease progression. Studies have also demonstrated that plasma p-tau231, similarly to plasma p-tau181, could identify clinical stages and neuropathology, with the advantage of increasing earlier than p-tau181 in response to early brain tau deposition, even before the threshold for Aβ PET positivity is reached [48]. Research on plasma p-tau231 and p-tau217 concludes that these biomarkers more effectively capture the earliest cerebral Aβ changes, even before the observable presence of Aβ deposits, thus indicating plasma p-tau231 and p-tau217 as promising biomarkers for preclinical AD [48].

## 4. Characteristics of tau Protein

Tau protein is encoded by the MT-associated protein tau (*MAPT*) gene located on human chromosome 17 at band position 17q21 [50,51,52,53]. It was discovered in 1975 [51,54,55]. The *MAPT* gene contains 16 exons [51,56,57], although exon 1, being a part of the promoter, and exon 14 under transcription but not translation [56,57]. Exons 9–12 play a crucial role, encoding four highly conserved imperfect repeats that make up the MT-binding domain of tau [56]. Uniquely, tau mRNA is transported to the proximal axon for translation, which facilitates the establishment of neuronal polarity [51].

Tau protein is divided into four functional domains: the N-terminal projection domain (NTPD), proline-rich regions (PRRs), MT-binding domain (MTBD), and C-terminal domain (CTD) [50,51,56]. The protein exists in six isoforms [52,53,55,56], which result from the alternative splicing of exons 2, 3, and 10 [51,52,53,56]. These isoforms include 3R0N, 3R1N, 3R2N, 4R0N, 4R1N, and 4R2N. The first three are collectively referred to as 3R isoforms, and the latter three as 4R isoforms. The number of ‘R’s indicates the number of MTBDs included in an isoform, and ‘N’ indicates the number of NTPDs [50,52,53,55,56]. The isoforms range from 352 to 441 amino acid residues in a chain [50,52,57]. In the fetal human brain, only 3R isoforms are expressed [50,51,53,56]. In the adult human brain, the expression of tau isoforms varies between regions; for example, the cerebellum has an elevated amount of 3R0N, whereas in the globus pallidus, 4R isoforms dominate [56]. However, physiologically, the ratio of 3R to 4R tau isoforms in the adult human brain should be approximately one to one [51,52,56]. An abnormal ratio of these isoforms is a characteristic feature of tauopathies such as AD [51]. This is why the alternative splicing of exon 10, which determines the type of protein (3R or 4R), is the subject of intensive scientific research [50].

Compared to other proteins, 2N4R, the longest human tau isoform present in the central nervous system (441 amino acid residues), has a relatively low proportion of hydrophobic amino acid residues, making tau generally a hydrophilic protein [56]. Tau is a natively unfolded protein that maintains a highly flexible conformation with a low content of secondary structure [56]. However, this does not preclude the possibility of folding through intramolecular interactions between differently charged domains [56]. MTBD consists of four repeating motifs, separated by flanking regions. Notably, the second and third repeats of the MTBD have a tendency to form a β-sheet structure [56].

Tau protein is described as a scaffolding protein [50]. Its main function is the stabilization of MTs in the distal portions of axons, which are essential for axonal transport systems. These systems ensure the transport of organelles and signaling molecules, making tau protein crucial for the proper functioning of neurons [51]. In that way, tau is also responsible for the dynamics of axonal growth cones [52]. Furthermore, some studies have reported tau’s interaction with actin, which influences actin polymerization and the interaction between actin filaments and MTs. Additionally, tau has other functions, such as binding to molecules like PSEN-1 and RNA [51].

The conformation of tau has a “paperclip” form, in which the CTD folds over MTBD and the NTPD folds back over the CTD, bringing the two termini in close proximity [56]. The association between CTD and NTPD is reduced when tau binds to MTs. NTPD projects away from MTs without binding to them directly; however, it plays a role in regulating MT dynamics by influencing the attachment and spacing between MTs and other cellular components. Additionally, the extreme N-terminal region of tau (residues 2–18) is involved in a signaling cascade that inhibits axonal transport in neurons [56]. However, the specific functions of NTPD are not yet well established, although it is hypothesized to influence tau distribution because the 0 N, 1 N, and 2 N isoforms exhibit distinct subcellular localizations in the mouse brain [56].

Tau protein can also be found in dendrites and at the post-synapse under both physiological and pathological conditions [50,51,53]. However, its physiological role in the mentioned compartments is less understood [50]. The binding of tau to MTs occurs through interactions between MTBD and α- and β-tubulin heterodimers [50] within specific pockets in the tubulin on the inner surface of MTs [51]. Because the MT surface is negatively charged, this binding is enhanced by the presence of positively charged PRRs [50,51]. The C-terminal domain likely plays a role in inhibiting tau polymerization [50].

Post-translational modifications, including phosphorylation, N- and O-glycosylation, ubiquitination, truncation, and oxidation, regulate the interaction between tau and MTs [50,55]. Among them, the most important role in the pathogenesis of tau is played by phosphorylation, which is directly related to the pathophysiology of AD [52].

## 5. Characteristics of Hyperphosphorylation Process of tau Protein

Phosphorylation is the primary post-translational modification regulating the binding of tau to MTs under physiological conditions. Approximately 85 putative phosphorylation sites have been predicted, with over 50 of these sites confirmed to be modified in tau [55]. Most of these phosphorylation sites are found in the C-terminal half of the amino acid sequence [55], although notable exceptions, such as Thr217 and Thr231, are situated in the N-terminal region [34].

In the physiological state, tau phosphorylation plays a crucial role, with phosphorylation at Ser396 and Ser404 stabilizing beta-catenin, rendering cells anti-apoptotic and providing neuronal protection [58]. This process also significantly contributes to adult hippocampal neurogenesis. Both aggregated and soluble tau found in the brain of AD patients show reactivity to antibodies detecting phosphorylated tau, as well as to an antibody reactive for non-phosphorylated tau following alkaline phosphatase digestion [55]. This has led to the conclusion that tau in its hyperphosphorylated form is associated with tau aggregation and toxicity, with a damaging effect on the brain [58]. In autopsied AD brains, tau phosphorylation level has been found 3–4 times higher than in healthy individuals [30], marking it as an early pathology in the development of AD [58]. Hyperphosphorylation of tau at sites such as Thr205, Thr231, Ser262, and Ser396 is known to attenuate endoplasmic reticulum stress and death-associated protein kinase-induced apoptosis [58]. It also reduces the affinity of tau for MTs, leading to the formation of highly toxic tau oligomers [58]. Critically, in the pathogenesis of AD, hyperphosphorylation leads to the dissociation of tau from MTs, contributing to the formation of NFTs, MT collapse, axon degeneration, and axoplasmic transport disorders [34]. This disrupts the distribution of neurotransmitters (including their synthesis, transport, release, and uptake), culminating in neurodegeneration [58].

**Figure 1:**
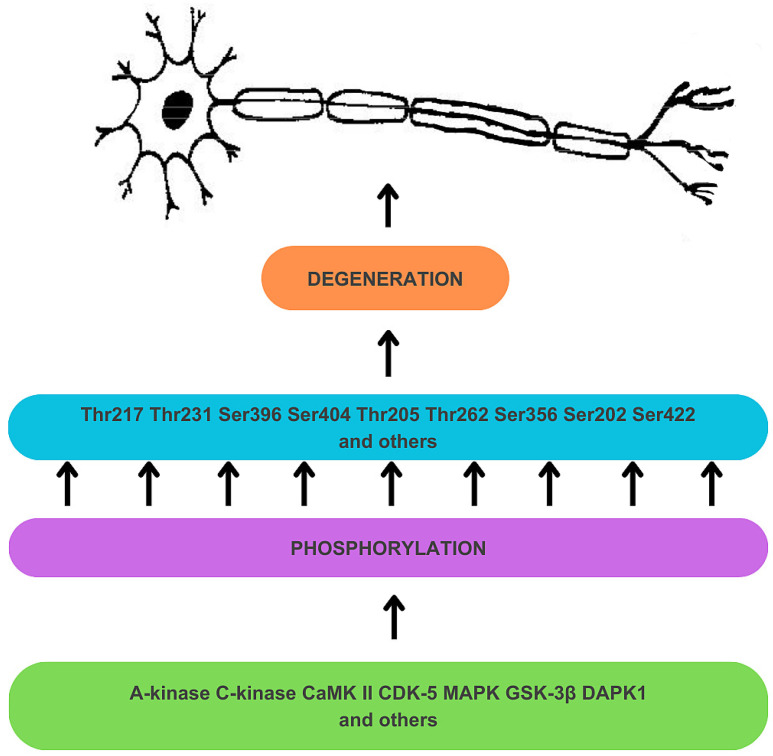
Mechanism of hyperphosphorylation of tau protein in the development of neuronal degeneration. Dysregulation of Ser/Thr kinases, including A-kinase, C-kinase, calmodulin kinase II (CaMK II), cyclin-dependent kinase 5 (CDK-5), mitogen-activated protein kinase (MAPK), glycogen synthase kinase-3β (GSK-3β), and death-associated protein kinase 1 (DAPK1) results in tau hyperphosphorylation, which contributes to tau dysfunction and neurodegeneration.

Hyperphosphorylation results from the deregulation of Ser/Thr kinases (see Figure 1) [34]. A-kinase, C-kinase, calmodulin kinase II (CaMK II), cyclin-dependent kinase 5 (CDK-5), mitogen-activated protein kinase (MAPK), and glycogen synthase kinase-3β (GSK-3β) are responsible for the phosphorylation of Thr 217 and Thr 231 sites, among many other sites on tau protein [34]. Tau protein undergoes prephosphorylation at sites such as Ser262/Ser356 by C-kinase, CaMK II, and CDK-5, making it more susceptible to subsequent phosphorylation by GSK-3β [34]. The prephosphorylation at Ser262/Ser356 may induce a conformational change in tau, enabling GSK-3β to recognize Thr231 and other sites more readily [34]. GSK-3β is also directly involved in the phosphorylation of Thr217 [59]. The process of tau hyperphosphorylation is further accelerated by Aβ, which mediates the activation of CDK-5 and GSK-3β [34]. Deregulation of kinase activities may also result from a disruption in the expression of non-coding RNA genes. For instance, decreased activity of miRNA-195 in knock-out rat models has been found to activate Cdk5/p25 signaling, promoting the phosphorylation of tau at Ser202, Thr205, Ser262, and Ser422 sites, and, crucially for this discussion, at the Thr231 site [58,60]. Furthermore, overexpression of death-associated protein kinase 1 (DAPK1) has been shown to increase tau protein stability and phosphorylation, including the Thr231 site [58,61].

Protein phosphatase 2A (PP2A) plays a central role in tau dephosphorylation and is implicated in AD’s pathogenesis [62]. PP2A accounts for the majority of tau phosphatase activity in the brain, and its activity is found to be reduced in AD, contributing to abnormal tau hyperphosphorylation and aggregation. In their review, Martin et al. [62] highlight the predominant role of PP2A in regulating tau phosphorylation sites, showing that disruptions in PP2A activity could significantly impact tau’s pathological phosphorylation in AD. The involvement of other phosphatases, such as protein phosphatase 1 (PP1), protein phosphatase 5 (PP5), and phosphatase and tensin homolog deleted on chromosome 10 (PTEN), although less pronounced than PP2A, still contributes to the complex regulation of tau phosphorylation and has been observed to be altered in AD as well. The balance between kinase and phosphatase activities determines the phosphorylation status of tau, and the disruption of this balance leads to the tau pathology observed in AD.

In summary, tau hyperphosphorylation is a multifactorial and complex process that plays a key role in the pathomechanism of tauopathies, including AD. Undoubtedly, further research is necessary, which may bring a breakthrough in the treatment and prevention of neurodegenerative diseases related to the abnormal function of the tau protein.

## 6. From tau Phosphorylation to Neurofibrillary Tangle Formation

Tau, as a ‘tubulin-associated unit’ and a protein constituting over 80% of MT-associated proteins, primarily plays a physiological role in bundling and stabilizing axonal MTs through tubulin polymerization, a process that is disrupted by phosphorylation [63,64,65]. Additionally, phosphorylation, particularly by GSK-3β, results in reduced tau transport along the axon due to diminished interaction with kinesin [64,66]. The primary pathway for tau phosphorylation involves protein kinases such as GSK-3β, cAMP-dependent protein kinase, MT affinity-regulating kinase 4, and CDK-5. The phosphorylation by the mentioned kinases causes the destabilization of MTs. It has been demonstrated that the phosphorylation of tau is inversely correlated with its association with MTs [63,64,65,66,67]. Moreover, the specific consequences of tau phosphorylation depend on the site of modification [65,68]. For example, in contrast to the majority of the sites, phosphorylation at Ser208 leads to an increase in tau’s affinity for binding MTs, but it does not reduce tau aggregation, as this modification is particularly found in late-stage NFTs [68]. Man et al. [65] found that phosphorylation at Ser289 and Ser293 leads to enhanced oligomerization and a change in the structure of the tau peptide chain from a helix to a coil [63]. Thus, this transformation to an unorganized structure may be a “driving force” for the aggregation of tau [65]. Unfortunately, the exact mechanism of NFT formation is not fully understood, and further studies on this subject are necessary [66,69]. Regardless of the exact mechanism, the diminished affinity for MTs through phosphorylation primarily leads to aggregation, which in turn plays a role in the formation of NFTs [64,65]. Furthermore, it has been stated in numerous studies that NFTs are present in AD pathology and are strictly correlated with neurodegeneration in the development of AD [63,64,65,66,67,68,70]. Higher concentrations of phosphorylated tau have been correlated with neuronal damage and death in the process of dementia. Emerging theories suggest that NFTs may have a role in AD that differs from the traditionally proposed one, with some suggesting a protective function [64]. Buée et al. [64] proposed that NFTs may have a protective role against toxic phosphorylated tau species during neurodegeneration. They argue that, while apoptosis takes 24 h to complete, the tau aggregation leading to cell death spans over 24 years. This suggests that NFTs might prolong the disease development by more than two decades before neurons ultimately succumb to the toxic effects of the protein’s accumulation [64]. Regardless of the actual role of NFTs, their strong correlation with phosphorylated tau and neurodegeneration remains undisputed, advancing the field towards the better detection of AD pathology.

## 7. P-tau Isoforms as AD Biomarkers

### 7.1. P-tau Isoforms Are Effective AD Biomarkers

The first step in challenging the status of p-tau181 as the ‘gold standard’ biomarker is to demonstrate that p-tau181 and other p-tau species exhibit the necessary accuracy and correlations to qualify as biomarkers in AD [71]. P-tau181 has been proven to accurately discriminate between Aβ and tau stages and correlate with their PET statuses [71]. It has been confirmed in multiple studies that other tau species, such as p-tau217 and particularly p-tau231, are as effective or even more effective for early AD detection than p-tau181 [72,73,74,75,76,77,78,79,80,81]. What makes p-tau variants useful biomarkers is their specificity for the so-called “biological AD”, which means a positive status for both Aβ PET (A+) and tau PET (T+). However, this correlation varies, as demonstrated by Therriault et al. [80], who, based on comparative analysis, found that p-tau biomarkers had a significantly higher correlation with Aβ PET than with tau PET. Accordingly, a study by Palmqvist et al. [82] states that the accumulation of Aβ promotes an increase in the production of soluble tau in neurons. This mechanism explains high levels of p-tau, especially p-tau217, in AD. Moreover, this mechanism is not present in other tauopathies. This finding has also been supported by Barthélemy et al. [79], who demonstrated an inverse correlation of the plasma p-tau217/tau217 ratio with the CSF Aβ42/Aβ40 ratio. This suggests that normal concentrations of Aβ are associated with reduced tau phosphorylation [79].

As it was previously mentioned, Aβ and p-tau show a strong correlation in AD pathogenesis. This also reflects the correlation in detecting the presence of Aβ with the higher levels of plasma p-tau217 [56]. Plasma p-tau217 mediates the association between accumulated Aβ and tau, and plasma p-tau217 may indicate the early accumulation of Aβ even before widespread tau aggregation occurs [56]. Other research studies report that p-tau217 and p-tau231 capture the earliest changes in cerebral Aβ imaged on PET [48] and that p-tau231 reaches abnormal levels with the lowest Aβ load [48]. To clarify, plasma levels of p-tau181 and p-tau217 are elevated in individuals with an A+T+ profile compared to A+T- and A−T-, and in A+T- compared to A−T- [81,83,84,85]. Importantly, the CSF exhibits a higher concentration of p-tau species, and as shown in the study by Barthélemy et al. [79], the p-tau/tau ratios are significantly higher in the CSF compared to the blood plasma. However, the use of blood-derived p-tau isoforms as AD biomarkers is still considered feasible because they reflect tau changes in the CSF. Moreover, peripheral phosphorylation of tau exhibits a distinct profile [79]. However, according to Palmqvist et al. [86], the accuracy of CSF and plasma biomarkers is similar in significance [86]. Plasma p-tau217 has been found to perform worse than its CSF counterpart [82]. However, due to the lower cost and the ease of testing, it may still serve as a viable biomarker in facilities with limited or no access to CSF or PET testing [82]. Blood serum is also considered a viable medium for measuring p-tau concentration, as its accuracy has been demonstrated to be similar to that of plasma [76].

Higher concentrations of p-tau181 and p-tau271 correlate with higher levels of AD neuropathological change (ADNC) and the *APOE* ε4 allele, which is directly involved in inherited AD [73,83,87]. However, the use of *APOE* ε4 as a prediction factor did not show a significant improvement in the effectiveness of the combination of plasma markers Aβ42/40 and plasma p-tau217 [88]. According to Woo et al. [84], p-tau181 and p-tau217 demonstrate the highest efficacy in differentiating T+ from T− patients. However, the accuracy of p-tau181 in determining tau PET status diminishes when considering only A+ patients, because only individuals with concurrent amyloid and tau pathologies can definitively be diagnosed with AD [84]. P-tau217 maintains a performance of over 85% in discriminating between T+ and T− even with the introduced change [84]. Additionally, p-tau217 correlates with common AD assessment scores, such as the Mini-Mental State Examination (MMSE) and clinical dementia rating (CDR), the scales typically used for evaluating a patient’s mental capabilities and potential neuropathology [84]. According to Janelidze et al. [89], considering the tau PET prediction, CSF p-tau217 correlates better than CSF p-tau181 with CSF Aβ42 and the retention of [18F] flortaucipir and [18F] flutemetamol, two radiopharmaceuticals used to visualize brain pathologies in AD on PET scans [89]. P-tau217 also is more accurate when it comes to the identification of patients with a pathological increase in [18F] flortaucipir binding [89]. The results from the article by Mundada et al. [90] also indicate a strong correlation between p-tau217 and [18F] flortaucipir. These findings confirm the superiority of p-tau217 over p-tau181 in tau PET prediction [89].

The performance of p-tau species depends on their clinical status. P-tau217 is considered a versatile biomarker as it accurately differentiates A+ from A− in both cognitively unimpaired (CU) and MCI groups. It has also been shown to increase with age [49,84,87]. Woo et al. [84] established thresholds for p-tau217 levels indicative of tau PET positivity: levels above 0.09 pg/mL (sensitivity = 0.91, specificity = 0.9, accuracy = 0.91) for the general population and levels above 0.14 pg/mL (sensitivity = 0.90, specificity = 0.72, accuracy = 0.79) for Aβ+ individuals. This distinction highlights a higher threshold for A+ individuals, suggesting that Aβ accumulation can lead to an increase in p-tau not necessarily associated with tau PET positivity [84]. Moreover, the study by Mattsson-Carlgren et al. [49] confirmed a similar pattern of p-tau217 changes in different stages of AD development, including CU, MCI, and AD conversion, with a higher baseline for A+ with a significant increase, and a stable baseline for A− and other dementia converters [49]. Analogously, Jonaitis et al. [91] provided evidence that baseline levels of plasma p-tau217 correlate with the trajectory of cognition: higher levels are associated with a steeper, more negative trajectory, while lower levels correlate with a more stable, flatter trajectory [91]. Furthermore, studies by Brickman et al. [73] and Yu et al. [92] showed that higher concentrations of plasma p-tau, specifically p-tau217, were correlated with posthumously confirmed AD [73,92].

The ability of a biomarker to differentiate AD from other neurodegenerative diseases is a crucial aspect of its effectiveness. The study by Yu et al. [92] indicated that p-tau217 is not associated with neurodegenerative diseases other than AD, with the exception of cerebral amyloid angiopathy (CAA). It has been shown that patients with both AD and CAA, of which the latter is also closely related to Aβ, have significantly higher levels of p-tau217 [92,93]. Importantly, plasma p-tau has been found to distinguish AD from primary age-related tauopathy (PART) [92]. High levels of p-tau217 and p-tau181 have been correlated with a greater likelihood of AD rather than PART.

From a practical standpoint, comparing the levels of tau and p-tau proteins in serum and blood plasma is critically important. In the study by Kac et al. [76], the diagnostic potential of p-tau231 and p-tau181 levels in serum and plasma for AD has been explored. Conducted across three cohorts with a total of 115 participants, the study revealed that p-tau levels in both serum and plasma were significantly higher in AD patients than in controls, indicating good diagnostic performance in serum. Notably, p-tau231 was found at lower concentrations in serum compared to plasma. Despite this, the strong correlation between p-tau levels in serum and plasma underscored the viability of serum as a practical alternative for AD diagnostics and research. These results endorse the use of serum in environments that prefer it to plasma, emphasizing its effectiveness for p-tau analysis. However, the necessity for further validation in independent cohorts and across different p-tau assays should be emphasized.

There are some limitations to the studies described above on the use of p-tau variants as biomarkers. It should be emphasized that the findings cannot be generalized to all individuals due to insufficiently diverse ethnic representation in the research groups [81,82,94]. The need to extend the research to unselected primary care populations should be also underlined [82]. Moreover, using plasma p-tau as a biomarker has some outstanding challenges such as the need for analytical guidelines, inter-laboratory method comparison and standardization, cut-off value generation and validation, and appropriate use criteria for clinical implementation [82,95]. The higher cost of mass spectrometry tests, which have shown the best performance compared to currently used immunoassays, has also been indicated [94].

### 7.2. Diagnostic Performance of Various p-tau Assays

Several trials have examined assays for measuring different p-tau species in order to use them as diagnostic tools in AD. The performance of various p-tau assays in differentiating AD patients is summarized in Table 1. The trial conducted by Bayoumy et al. [96] utilized six different plasma immunoassays: the p-tau217 assay from Lilly Research Laboratories, Indianapolis, IN, USA (Lilly); p-tau181 assay from Lilly; p-tau181 assay from Adx Neurosciences, Ghent, Belgium (ADx); p-tau231 assay from ADx; p-tau181 assay from Quanterix, Billerica, MA, USA (Quan); and p-tau231 assay from University of Gothenburg, Gothenburg, Sweden (UGot). The study confirmed that p-tau levels were significantly higher in AD patients compared to controls. The increase ranged from 4.1-fold for p-tau217 Lilly to 1.3-fold for p-tau231 ADx. Notably, the p-tau217 Lilly assay, all p-tau181 assays, and the p-tau231 UGot assay demonstrated high diagnostic accuracy for AD [96]. A post hoc comparison of the receiver operating characteristic (ROC) analysis of the area under the curve (AUC) indicated that p-tau181 ADx and p-tau217 Lilly both performed well, outperforming p-tau181 Quan. Moreover, p-tau217 Lilly surpassed p-tau231 UGot in performance, with p-tau181 ADx showing a similar trend. Additionally, both p-tau217 Lilly and p-tau181 ADx had better performance than p-tau181 Lilly. The p-tau231 ADx assay was the least effective, being outperformed by all other assays.

The study by Janelidze et al. [97] compared 10 assays: the p-tau181 assay from Washington University, Washington, DC, USA (WashU); p-tau181 ADx; p-tau181 Lilly; p-tau181 UGot; p-tau181 assay from Fujirebio Inc, Tokyo, Japan (Fuji); p-tau181 S-Plex immunoassay from Meso Scale Discovery, Rockville, MD, USA (Splex); p-tau217 WashU; p-tau217 Lilly; p-tau217 assay from Janssen Research and Development, Raritan, NJ, USA (Janss); and p-tau231 UGot. The results were in line with other findings, suggesting an increase in various p-tau species in A+ individuals compared to controls [97]. In this study, the best-performing assay was p-tau217 WashU, a mass spectrometry (MS)-based assay, followed by two immunoassays, namely p-tau217 Lilly and p-tau217 Janns [97]. On average, p-tau217 assays achieved higher AUCs than p-tau181 assays. In summary, the fold increase in p-tau species ranged from 1.2 to 3.6 [97]. When it comes to distinguishing MCI patients with progression to AD from those without AD, p-tau217 WashU was the best, followed by p-tau217 Lilly [97]. However, p-tau217 Janss, p-tau181 ADx, and p-tau181 WashU were not significantly worse than p-tau217 Lilly in that case, whereas other assays performed with significantly lower AUCs [97]. P-tau217 WashU performed with a higher AUC than other assays, which corroborated the superiority of MS-based assays for p-tau quantification [97]. It is worth noting that the concentration of p-tau217 in the CSF is approximately 5 times lower than that of p-tau181, which makes this quantification more difficult [99]. However, it should be concluded that p-tau217 has superior accuracy as a biomarker for AD compared to p-tau181 [97]. Expectedly, CSF p-tau measurements appeared to have AUC values unmatched by the corresponding plasma assays [97]. The strongest correlation was observed in the case of p-tau217 WashU. P-tau217 Lilly revealed a significant difference between the CSF and plasma correlation coefficients [97]. Other assays had moderate or weak correlations [97]. P-tau217 Lilly was found to be the immunoassay with the highest AUC; however, this was not the only assay with potentially useful accuracy [97]. P-tau217 Janss and p-tau181 ADx performed comparably in indicating Aβ deposition and possible progression to AD [97]. Additionally, a post hoc analysis revealed a significant increase in plasma p-tau217 levels in MCI to AD progressors in comparison to A+ non-progressors. This characteristic was not demonstrated by p-tau181 or p-tau231 [97].

The study by Ashton et al. [81] examined the plasma p-Tau217 immunoassay from ALZpath Inc., Carlsbad, CA, USA (ALZpath), and concluded that it predicts abnormal Aβ-PET and CSF Aβ42/40 with high accuracy. These results suggest that the performance of plasma p-tau217 ALZpath is comparable to that of CSF measurements and superior to that of brain atrophy assessments. Furthermore, its properties and accuracy significantly surpass those of other plasma biomarkers. In a long-term perspective, p-tau217 ALZpath levels tend to increase only when the Aβ accumulation is present and enhance further if tau pathology coexists [81].

Another study by Mielke et al. [100] found that, for the Meso Scale Discovery (MSD) p-tau181, MSD p-tau217, and single-molecule array (Simoa) p-tau231 platforms, p-tau levels were significantly higher in T+ compared to A− and T− groups. The study also highlighted that MSD p-tau217 exhibited the highest fold change and diagnostic performance among all evaluated platforms. Conversely, Simoa p-tau231 showed the lowest performance.

In the study by Ashton et al. [98], 18 immunoassays, including 9 plasma-based and 9 CSF-based, were examined. The plasma biomarker group included p-tau181 ADx, p-tau217 Janss, p-tau217 Lilly, p-tau181 Lilly, p-tau231 UGot, p-tau181 UGot, p-tau181 Quanterix, t-tau Lilly, and p-tau231 ADx. The CSF group comprised the CSF counterparts of these plasma assays. All biomarkers showed a significant increase in the AD CSF profile group compared to the non-AD (NAD) group, although the extent of change varied. The largest differences in plasma, categorized as ‘large’, were observed for p-tau181 Lilly, p-tau217 Lilly, p-tau181 ADx, p-tau217 Janss, and p-tau231 UGot. All CSF biomarkers were categorized as having a ‘large’ effect size. Further analysis revealed that the five mentioned plasma p-tau assays significantly outperformed the other plasma p-tau biomarkers in terms of differentiation accuracy. The study also found strong correlations between CSF and plasma immunoassays of the same type for p-tau181 ADx, p-tau217 Janss, p-tau181 Lilly, and p-tau217 Lilly. However, the discrimination accuracy of p-tau231 ADx, t-tau Lilly, p-tau181 Quanterix, and p-tau181 UGot was significantly weaker in plasma than in CSF.

In light of the aforementioned assays as a whole, a pattern emerges—tests from the same manufacturer for p-tau217 show higher AUC and fold increase than for p-tau181. These results lay a foundation for the conclusion that p-tau217 seems to be more accurate than p-tau181 and might be widely used as an AD biomarker.

### 7.3. P-tau217 Proves to Be Superior to p-tau181 and Other Isoforms

After proving the usefulness of p-tau-based biomarkers, several studies have focused on finding the most effective one. Generally, P-tau217 has been found to be a better option. Ashton et al. [78] and Barthélemy et al. [101] reported that both CSF and plasma p-tau217 levels increased 6-fold in CU A+ individuals compared to CU A−, and in AD compared to NAD. This increase was significantly higher than in the case of other p-tau biomarkers. For instance, plasma p-tau181 exhibited only a 1.3-fold increase. Moreover, with CSF p-tau181, a decrease in performance was observed when distinguishing MCI from NAD or CU, an issue not observed with CSF p-tau217 [78]. Additionally, several studies have proclaimed that plasma p-tau217 and p-tau181 are elevated in AD groups compared to controls [73,78,102]. Thijssen et al. [102] presented that p-tau217 increased by 4.4-fold and p-tau181 by 2.8-fold in clinical AD compared to healthy people. In an AD group compared to a frontotemporal lobar degeneration (FTLD) group, plasma p-tau217 and p-tau181 levels were elevated. P-tau217 demonstrated a higher fold change than p-tau181 in distinguishing AD from clinical FTLD-spectrum disorders (3.5-fold vs. 2.4-fold), FTLD-tau (4.1-fold vs. 2.8-fold), and FTLD-TDP (5.6-fold vs. 3.7-fold) [102]. In addition to demonstrating the specificity of p-tau species for AD and the superiority of plasma p-tau217 in that matter, the study proved that p-tau can be used to differentiate AD from FTLD, again with p-tau217 clearly leading in terms of maximum accuracy [102]. Moreover, Barthélemy et al. [79] used the p-tau/t-tau ratio to normalize tau variability and evidenced that p-tau217/tau217 exhibited an almost 9-fold increase compared to p-tau181/tau181 (+220% vs. +25%). Moreover, p-tau217 reached a greater increase in Aβ+ than p-tau181 (from +230% to +340% vs. from +60% to +80%) [79]. These findings support the thesis that the p-tau217/tau217 ratio reasonably distinguishes Aβ+ and Aβ− CU, whereas the p-tau181/tau181 ratio is not so accurate [79]. Interestingly, Salvadó et al. [103] indicated that p-tau217, used in the study along with four other biomarkers for successfully staging AD, continued to increase even after reaching its threshold of positivity. However, according to Barthélemy et al. [99], there is a negative correlation between both mentioned-above ratios and neurodegeneration measured by MRI and longitudinal cognitive decline. This suggests a reversal in the rate of phosphorylation during the disease progression. Thijssen et al. [102] also found that plasma p-tau217 has slightly higher AUC than p-tau181 for differentiating T+ and T−. However, there was no significant difference in distinguishing positive tau-PET between p-tau217 and p-tau181 in subgroups of MCI and corticobasal syndrome [102]. These findings were not observed in A− participants but were present in A+ patients [78,102]. In summary, plasma p-tau217 has been found to perform better than p-tau181 in multiple ways such as a higher fold increase and therefore a higher AUC for differentiating AD from FTLD, A+ from A−, and T+ from T−, although the magnitudes of differences varied between studies. However, in several studies, the results have indicated no significant differences between p-tau217 and p-tau181 in terms of distinguishing Aβ-PET or tau-PET [78,87,89,102].

Nevertheless, Barthélemy et al. [101] suggested that p-tau217 may be significantly better at the differentiation of AD from NAD because of an overlap in the distribution of p-tau181 values between NAD and AD. The aforementioned overlap did not exist for p-tau217. P-tau181 levels in several conditions classified as NAD, such as FTLD, Lewy body dementia, and progressive supranuclear palsy, have been found to reach the range seen in AD, whereas p-tau217 levels in these cases have been found to be different to those in AD. These findings support the superior differential abilities of p-tau217. On the contrary, according to Palmqvist et al. [86], there were no significant differences between plasma p-tau217 and p-tau181 in the predictive accuracy for progression to AD within 4 years. They concluded that p-tau and its variants are highly accurate in predicting AD, and their predictive power can be enhanced by combining them with brief cognitive tests of memory and executive function, as well as the *APOE* genotype [23]. They also noted that p-tau217 outperforms clinical diagnostic predictions of AD, which include medical history, memory assessment, and MRI and CT imaging [23]. This underscores the significant value of p-tau biomarkers in supporting clinical assessments in the diagnosis of AD.

According to the study by Mattsson-Carlgren et al. [104], p-tau217 is the most potent individual biomarker in both plasma and CSF for predicting modified Preclinical Alzheimer Cognitive Composite (mPACC) and MMSE slopes, with the levels in CSF showing marginally better performance than those in plasma. Yu et al. [92] also stated that p-tau217 offers higher accuracy for differentiation than p-tau181. Moreover, according to Janelidze et al. [88], p-tau217 can be outperformed by a combination of plasma Aβ42/40 with plasma p-tau217; however, this combination does not always produce a significantly better result than p-tau217 alone. Another study by Palmqvist et al. [105] established a combination of three plasma biomarkers, namely Aβ42/40, p-tau181, and *APOE*. In this combination, p-tau181 was interchangeable with p-tau217 with no difference in AUC. However, the study ultimately identified plasma p-tau181 as the biomarker with the highest predictive value for AD.

The results from a study by Yu et al. [92] suggested that plasma p-tau217 was more strongly correlated with Aβ plaques and tau tangles than plasma p-tau181. Additionally, the risk of progressing to AD nearly quadruples with every fold increase in plasma p-tau217 levels, whereas it only triples with each fold increase in plasma p-tau181 [92]. Salvadó et al. [93] showed that plasma p-tau217 exhibited the highest correlations with plaques and tangles of all biomarkers tested, including plasma p-tau181, which significantly outclassed other biomarkers, except Aβ42/40 and glial fibrillary acid protein (GFAP) for plaques and tangles, respectively. However, it is worth noting that none of these biomarkers came close in terms of correlation with both plaques and tangles [93]. The strong association between p-tau217 and both Aβ plaques and tau tangles has been further confirmed by Mattsson-Carlgren et al. [106]. These findings make p-tau217 a versatile and accurate biomarker, with the ability to assess the status of both amyloid and tau at once [93]. In the same study, the authors developed parsimonious models for detecting amyloid plaques and neurofibrillary tangles. The models for plaques consisted of plasma p-tau217 and Aβ42/40, and the models for tangles included only plasma p-tau217 [93]. The efficacy of plasma p-tau217 as a sole biomarker, surpassing combinations of biomarkers, further corroborates its superiority [93].

**Figure 2:**
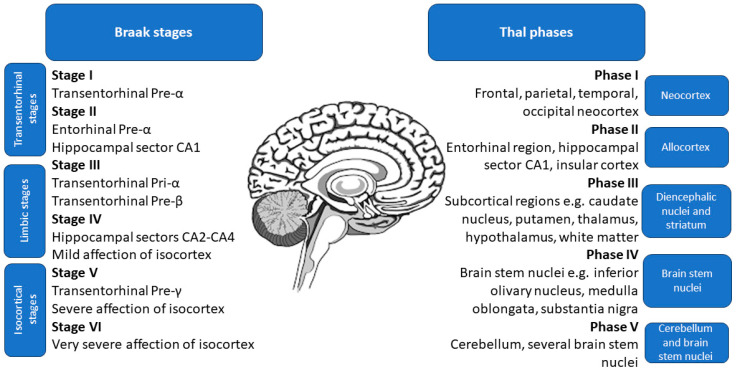
Location of pathological changes in Braak stages and Thal phases, used to classify the brain pathology in Alzheimer’s disease assessed during an autopsy.

The study by Therriault et al. [107] suggested that p-tau217 exhibits a significantly smaller degree of change between CSF and plasma levels compared to p-tau181 and p-tau231, with an effect size of 84% instead of a mere 50%. Additionally, the overlap of CSF and plasma p-tau217 levels was similar, making it much more accurate than those of p-tau181 and p-tau231. Moreover, the fold change of plasma p-tau217 was higher than in the case of the CSF p-tau181 and p-tau231, further corroborating its high utility [107]. The study also found that the agreement on positivity between CSF and plasma variants was highest for p-tau217, at 88.5% (58.7% both negative, 29.8% both positive, and 11.5% discordant), while p-tau181 had a lower agreement rate of 74.7% (55.1% both negative, 19.6% both positive, and 25.3% discordant). Notably, none of the discordant results for p-tau217 occurred in patients with CDR of 1 or higher, or with positive tau-PET [107]. Plasma p-tau217 had higher accuracy for identifying Aβ PET and biological AD (Aβ and tau PET positivity) than plasma p-tau181 and plasma p-tau231, with not significantly different results from CSF variations of these markers [107]. Moreover, according to Montoliu-Gaya et al. [108], plasma p-tau217 had the highest accuracy for differentiating Aβ status. Accordingly, a study by Horie et al. [109] states that CSF p-tau217/tau217 ratio had the strongest correlation with Aβ PET. Post hoc comparisons revealed that p-tau217 values were significantly elevated in subjects at advanced stages of brain pathology assessed during an autopsy, namely Thal phases 4–5, Braak stages 5–6, and The Consortium to Establish a Registry for Alzheimer’s Disease (CERAD) frequent scores [102,110,111,112]. The specific locations of pathological changes in Thal phases and Braak stages are shown in Figure 2.

The superiority of p-tau217 as an AD biomarker is strengthened by the findings of Salvadó et al. [93] regarding the prediction of ADNC, CERAD classification, and Braak staging. According to their findings, p-tau217 achieves the highest accuracy for CERAD classification as an individual biomarker, comparable only to the Aβ42/40 ratio, and is not significantly inferior for CERAD classification and ADNC prediction, for which its combination with Aβ42/40 performed best. It also attained the highest accuracy for Braak staging, with only GFAP being similarly significant. According to Montoliu-Gaya et al. [108], plasma p-tau217 allows for distinguishing Braak I–IV from V–VI with decent accuracy [108]. Leuzy et al. [113] created a parsimonious model, using plasma p-tau217 and tau PET, which was associated with an annual change in the radioligand [^18^F]RO948 standardized uptake value ratio (SUVR) and could facilitate the recruitment for clinical trials on AD.

Interestingly, Salvadó et al. [93] suggested the use of the p-tau217/Aβ42 ratio as a novel biomarker. This ratio outperforms other parameters in predicting the presence of plaques and tangles and shows significant differences between the absence and low levels of ADNC.

Overall, the results of the described studies indicate p-tau217 as the best-performing tau biomarker, and even the best of all available biomarkers. CSF p-tau217 has the highest accuracy, followed by CSF p-tau181 and plasma p-tau217, but their comparison gives ambiguous results. Few studies indicate that plasma p-tau217 may be more effective than CSF p-tau181, but there is not much evidence to definitely prove this.

### 7.4. Longitudinal Effectiveness and Change in p-tau217 Levels

A study by Mattsson-Carlgren et al. [49] focused on long-term changes in levels of plasma p-tau217 and their diagnostic value. According to the article, a longitudinal increase in p-tau217 was associated with cognitive decline in both CU and MCI, also within the subgroups of A+ participants [49]. Additionally, p-tau217 levels were linked to faster atrophy of the temporal cortex and hippocampus in A− CU, A+ CU, and the overall MCI group, although this correlation was not significant in the Aβ+ MCI subgroup. As proven by Ashton et al. [114], plasma p-tau217 levels increased over time in the preclinical and early clinical stages of AD but remained stable in control groups, including A− CU, A− MCI, and MCI that did not convert to AD. Over time, longitudinal increases in plasma p-tau217 levels were significantly associated with worsening MMSE and mPACC scores, impaired delayed recall memory, and accelerated cortical thickness atrophy over 6 and 8 years. Notably, p-tau217 was the only biomarker significantly linked to cognitive decline, including Aβ+ CU individuals, while p-tau181 demonstrated only modest performance [114]. Moreover, according to Suárez-Calvet et al. [74], the commonly used mid-region p-tau181 and t-tau in CSF were less useful than CSF N-terminal p-tau181, N-terminal p-tau217, and mid-region p-tau231, which showed more evident increases at the onset of AD. These markers reached higher levels before Aβ, the current gold standard, reached its threshold. Furthermore, in early AD, the annual rate of increase in p-tau217 in entorhinal PET SUVR was higher among individuals with higher baseline levels compared to those with lower levels [85]. Additionally, longitudinal increases in p-tau217 were correlated with the burden of plaques and tangle load, a correlation not found for p-tau181 [93]. Overall, these results demonstrated that the longitudinal increase in p-tau217 is significantly correlated with AD development.

### 7.5. P-tau231 Is Probably the Earliest AD Biomarker

An important part of the treatment of neurodegenerative disorders is their early detection. In several studies, p-tau231 has shown exceptionally high accuracy in early-stage AD. Yakoub et al. [83] proved that p-tau231 levels increased in A+T+ individuals compared to A− T−, confirming its specificity for AD pathology. P-tau231 levels increased in AD, measured in CSF, plasma, and serum [76]. The increase in its levels ranged from 2-fold in plasma and serum to up to 5-fold in CSF [76]. Lilek et al. [115] showed evidence that p-tau231 primarily accumulates at the postsynaptic density, and this occurs in an early, often presymptomatic stage of the disease. The study of Montoliu-Gaya et al. [108] indicated that p-tau231 increased significantly along the AD continuum and that its levels did not increase in MCI and demented patients without AD pathology. Furthermore, its levels did not significantly elevate after Braak III-IV, supporting the hypothesis that p-tau231 increases early on and that the rate of this increase then slows down.

Plasma p-tau231 shows a strong correlation with Aβ PET measured using the radioligand [18F]AZD4694, with a particularly strong association in the precuneus, frontal cortex, and striatum [44,77]. Interestingly, in CU individuals, the relationship between plasma p-tau231 and Aβ PET is observed in both A+ and A− groups. However, in cognitively impaired patients, the correlation between plasma p-tau231 and Aβ is limited to A+ individuals only [50]. Additionally, plasma p-tau231 accurately predicts a 1-year decline in MMSE scores and hippocampal atrophy [44].

Plasma p-tau231 demonstrates superior accuracy in distinguishing between CU A+ and A− individuals compared to plasma p-tau181, plasma p-tau217, and the Aβ42/40 ratio, showing significantly better performance than other plasma biomarkers, with the exception of Aβ42/40 [44,74,114]. However, it has lower accuracy in differentiating between elderly CU and MCI Aβ+ individuals, highlighting its increase in the preclinical stages of AD [44]. Moreover, p-tau231 is more effective at distinguishing elderly CU Aβ+ from MCI Aβ–, but less effective in separating CU Aβ+ from AD, supporting the idea that p-tau231 levels rise in the early stages of the disease [44,78]. Levels of p-tau231 progressively increase from CU in young individuals to elderly, MCI, and AD stages, and p-tau231 can differentiate AD from both young and elderly CU individuals but not from MCI [44].

Studies by Ashton et al. [44] and Milà-Alomà et al. [48] indicated that plasma p-tau231 levels increased earlier than plasma p-tau181 in relation to Aβ deposition load and before Aβ reached its positivity threshold. Similar observations were made for the CSF levels of these biomarkers [78]. However, plasma results suggest that p-tau231 may better characterize the earliest changes in AD. This is also supported by the fact that p-tau231 levels plateau at later stages of the disease, while p-tau217 and p-tau181 levels continue to increase with higher Aβ burden [114].

The research conducted by Smirnov et al. [116] and Ashton et al. [44], regarding plasma p-tau231, revealed that it increased and correlated with Aβ PET, often before Aβ PET showed positivity. This explains its earlier significant increase compared to plasma p-tau181 and its ability to detect tau accumulation, which plasma p-tau181 did not exhibit. Furthermore, according to the study of Therriault et al. [80], CSF p-tau231, CSF p-tau217, and plasma p-tau231 showed a strong correlation with Aβ PET.

The performance of CSF p-tau231 as a predictor of A+ is superior to that of p-tau181 and is as significant as that of CSF p-tau217 and CSF Aβ42/40 [78]. Studies by Ashton et al. [78] and Smirnov et al. [116] supported the finding that p-tau231 is the first p-tau marker to reach abnormal levels within the pre-Aβ phase, making it a valuable tool for the early detection of AD pathology.

Furthermore, p-tau231 is quite accurate in predicting tau PET, with a 76% concordance rate. However, a study by Tissot et al. [77] noted that 20% of patients had a p-tau231+/tau PET- result, leading to the conclusion that an increase in p-tau231 levels begins well before tau PET reaches the threshold of positivity. Similarly to CSF concentrations, plasma p-tau231 showed differences at earlier Braak stages, while plasma p-tau181 showed larger differences at later Braak stages [116,117]. Compared to CSF concentrations, plasma p-tau231 does not reach a plateau in the later stages of AD; thus, it has a significantly lower performance in identifying A+T+ individuals [107,117].

In summary, p-tau231 is highly correlated with Aβ pathology and demonstrates an early increase, often in the pre-clinical stage of the disease, even before Aβ PET positivity is detected. However, its accuracy and utility decrease in later stages, as it does not differentiate between advanced stages, though it can clearly distinguish between early and later stages. Together, these features make p-tau231 a valuable complementary biomarker to p-tau217 as an early indicator of emerging AD pathology.

## 8. Discussion

The best-known physiological role of the tau protein, as the main MT-associated protein and the so-called scaffolding protein, is MT stabilization [50,63,64,65]. However, tau has been found to have a multitude of functions, including maintaining structural integrity, axonal transport, and signaling within and between neurons [51,56]. It can be assumed that this multifunctionality of the tau protein makes it one of two main proteins involved in AD pathology [34,58,59,60,61,63,64,65,66,67,68,69,70]. Tau hyperphosphorylation seems to significantly participate in the process of aberrant assembly and functioning of tau and, consequently, in the development of neurodegeneration. This phosphorylation can take place in different loci. Thus, the levels of several p-tau variants may indicate the state of the disease or even predict it, as well as differentiating AD from other neurodegenerative disorders [49,71,73,74,75,76,77,78,79,80,81,82,83,84,86,87,89]. In this narrative review, we have tried to gather and organize studies that, through experimentation, achieved results regarding the efficacy and utility of p-tau species as biomarkers for AD. The review provides a broad overview of the subject of p-tau species as AD biomarkers, especially p-tau217 and p-tau231, both in CSF and plasma. This summary of the current state of knowledge may inspire future research by identifying known facts and previously unexplored areas, indicating an urgent need for further research on AD. The fundamental purpose of AD research is to develop methods to detect and treat the disease [71,73,74,75,76,77,78,79,80,82,83,84,85,86,87,88,89,91,92,93,96,97,98,99,100,101,102,103,104,105,107,108,109]. This goal could be achieved by deepening our understanding of tau’s role in AD pathology, including identifying the feasibility of using p-tau species as biomarkers and potential therapeutic targets. Achieving these goals is difficult due to the late and expensive detection of AD, which impedes further research and requires the development of more accurate, less expensive, and easier-to-use markers in AD.

An important issue, which also requires further research, is the impact of disease comorbidities on tau changes in AD patients. Comorbidities seem to play a significant role in influencing the detection of tau and p-tau biomarkers, which is crucial for AD diagnostics. Studies indicate that systemic health conditions such as diabetes mellitus, kidney disease, and hypertension, along with demographic factors, may notably affect these biomarkers. Martínez-Dubarbie et al. [118] revealed renal function’s impact on plasma Aβ levels and the association of hypertension with increased p-tau181 levels. Similarly, Pan et al. [119] demonstrated that demographic factors, especially age, and comorbidities like cardiovascular and metabolic disorders affect p-tau181 plasma levels in cognitively normal individuals. Zenuni et al. [120] further validated the significant linkage between comorbidities related to heart, vascular disorders, and diabetes mellitus and CSF biomarkers for neurodegeneration, including tau and p-tau. Furthermore, Ossenkoppele et al. [121] investigated tau PET status across a broad cohort, identifying a high tau PET positivity in AD dementia and MCI with Aβ+ status, notably lower in non-AD and CU groups. Key predictors of tau PET positivity across these studies included younger age, lower MMSE scores, and reduced cortical thickness in AD-related conditions, highlighting the complex interplay between systemic health, tau pathology, and AD diagnostics. These findings underscore the importance of considering both comorbidities and demographic factors for the accurate interpretation of AD biomarkers, spotlighting the complex relationship between systemic health conditions and neurodegenerative disease markers.

Interestingly, blood levels of tau and p-tau differ significantly among individuals with cognitive impairments, traumatic brain injury (TBI), or COVID-19. The study by Dang et al. [122] emphasizes the significant correlation between tau accumulation and cognitive decline, as well as neuropsychiatric symptoms, showcasing its diagnostic superiority over Aβ for distinguishing AD patients from cognitively normal controls. Notably, tau PET imaging demonstrates greater accuracy than Aβ in this differentiation, with pronounced tau deposition in the inferior temporal lobes strongly associated with the severity of cognitive impairments. These results indicate that tau could act as a more sensitive and specific biomarker for both the diagnosis of AD and the tracking of cognitive decline’s progression, underscoring its value in clinical practice. The study by Rubenstein et al. [123] provides crucial insights into how blood levels of tau and p-tau differ among individuals with TBI. Its key findings indicate a significant increase in both t-tau and p-tau levels in serum and CSF within the first five days post-TBI, with a notably sharper rise in p-tau, suggesting a shift towards tau hyperphosphorylation. This hyperphosphorylation, marked by a substantial increase in the p-tau/t-tau ratio, points to an early and sustained alteration in tau dynamics post-injury. The study also reveals that higher chronic mean levels of p-tau, rather than t-tau, are associated with greater disability and worse outcomes up to 12 months post-TBI. This emphasizes the distinct and critical role of p-tau in the aftermath of TBI and its potential as a prognostic marker for long-term recovery and the development of tauopathies. The review by Sfera [124] explores the role of tau and p-tau blood levels in cognitive impairments among individuals with long COVID-19, also referred to as the post-acute sequelae of SARS-CoV-2. The authors suggest that chronic inflammation, triggered by viral remnants in specific body sites, results in the formation of pathological syncytia involving microglia and astrocytes. This, in turn, facilitates the seeding of hyperphosphorylated tau within the brain. This process is further exacerbated by the virus-induced increase in the permeability of the blood–brain barrier, enabling substances that can induce tau hyperphosphorylation, such as microbial components, to infiltrate the brain. Consequently, the review underscores the potential significance of blood levels of phosphorylated tau as biomarkers for cognitive dysfunctions in long COVID-19 patients, thereby establishing a link between SARS-CoV-2 infection and neurodegenerative processes.

## 9. Conclusions

Alzheimer’s disease remains an unresolved challenge in medicine. Undoubtedly, successful biomarkers could significantly accelerate the process of recruiting trial participants for researchers looking for effective therapies. Through a comprehensive analysis, this review endeavored to provide insights into the pathophysiological significance of p-tau217 and p-tau231 as biomarkers in AD progression and their prospective role in enhancing diagnostic protocols, guiding therapeutic interventions, and potentially serving as targets for future treatments. While studies generally agree on the superiority of p-tau217 as the most effective tau isoform and overall AD biomarker, following studies are essential for its implementation and the identification of other potential early-detection biomarkers. Nevertheless, further research on plasma biomarkers is warranted, as they appear to be the future of AD detection and potential early screening. The development of specific assays for p-tau measurement, which would enable easy clinical application of the discussed biomarkers, seems to be of first importance. We believe that this review will be useful for researchers, clinicians, and students eager to explore the subject of state-of-the-art AD biomarkers.

## Figures and Tables

**Table 1:** Diagnostic performance of various p-tau assays in distinguishing Alzheimer’s disease (AD) patients from healthy controls [96], normal and abnormal Aβ42/40 ratio in the cerebrospinal fluid (CSF) [97], and non-AD (NAD) patients [98]. Data are shown as median and interquartile range (in brackets).

Assay	Fold Increase in CSF	AUC (95% CI) in CSF	Fold Increase in Plasma	AUC (95% CI) in Plasma	Ref.
p-tau 181 Lilly	-	-	1.8	0.938 (0.872–1.000)	[96]
	-	-	1.2–1.4	0.759 (0.676–0.841)	[97]
	3.29	0.95 (0.91–0.98)	2.59	0.91 (0.86–0.96)	[98]
p-tau217 Lilly	-	-	4.1	0.995 (0.987–1.000)	[96]
	-	-	2.0	0.886 (0.827–0.944)	[97]
	7.18	0.98 (0.96–1.00)	3.27	0.94 (0.90–0.98)	[98]
t-tau Lilly	1.97	0.85 (0.79–0.90)	1.36	0.73 (0.65–0.81)	[98]
p-tau181 ADx	-	-	2.9	0.988 (0.969–1.000)	[96]
	-	-	1.8	0.841 (0.768–0.913)	[97]
	4.77	0.96 (0.93–0.98)	3.48	0.94 (0.91–0.97)	[98]
p-tau231 ADx	-	-	1.3	0.719 (0.607–0.831)	[96]
	3.59	0.93 (0.88–0.97)	1.39	0.66 (0.58–0.74)	[98]
p-tau181 UGot	-	-	1.2–1.4	0.743 (0.652–0.833)	[97]
	2.1	0.94 (0.90–0.97)	1.38	0.80 (0.73–0.87)	[98]
p-tau231 UGot	-	-	1.5	0.943 (0.896–0.991)	[96]
	-	-	1.2–1.4	0.784 (0.703–0.864)	[97]
	-	0.91 (0.87–0.95)	1.95	0.88 (0.83–0.93)	[98]
p-tau181 WashU	-	-	1.2–1.4	0.835 (0.765–0.906)	[97]
p-tau217 WashU	-	-	3.6	0.947 (0.907–0.987)	[97]
p-tau181 Fuji	-	-	1.2–1.4	0.694 (0.604–0.784)	[97]
p-tau181 Splex	-	-	1.2–1.4	0.642 (0.533–0.751)	[97]
p-tau181 Quanterix	-	-	1.9	0.936 (0.885–0.987)	[96]
	5.02	0.96 (0.93–0.99)	1.66	0.80 (0.73–0.87)	[98]
p-tau217 Janss	-	-	2.7	0.858 (0.795–0.920)	[97]
	8.53	0.98 (0.96–1.00)	5.22	0.96 (0.93–0.99)	[98]

Abbreviations: AUC—area under the curve; CI—confidence interval; p-tau181—tau phosphorylated at threonine-181; p-tau217—tau phosphorylated at threonine-217; p-tau231—tau phosphorylated at threonine-231. The origin of the p-tau assays: ADx—ADx Neurosciences, Ghent, Belgium; Fuji—Fujirebio Inc, Tokyo, Japan; Janss—Janssen Research and Development, Raritan, NJ, USA; Lilly—Lilly Research Laboratories, Indianapolis, IN, USA; Quanterix—Quanterix Corp., Billerica, MA, USA; Splex—S-Plex immunoassay from Meso Scale Discovery, Rockville, MD, USA; UGot—University of Gothenburg, Gothenburg, Sweden; WashU—Washington University, Washington, DC, USA.

## Abbreviations

Aβ	amyloid β
AD	Alzheimer’s disease
ADNC	AD neuropathological change
APOE	apolipoprotein E
APP	amyloid protein precursor
AUC	area under the curve
BACE-1	β-secretase
BMI	body mass index
BOLD	resting blood oxygen level-dependent imaging
CAA	cerebral amyloid angiopathy
CAMK II	calmodulin kinase II
CDK-5	cyclin-dependent kinase 5
CDR	clinical dementia rating
CERAD	The Consortium to Establish a Registry for Alzheimer’s Disease
CSF	cerebrospinal fluid
CTD	C-terminal domain
CU	cognitively unimpaired
FDG-PET	fluorodeoxyglucose-18 positron resonance imaging
fMRI	functional magnetic resonance imaging
FTLD	frontotemporal lobar degeneration
GFAP	glial fibrillary acid protein
GSK-3β	glycogen synthase kinase-3β
MAPT	microtubule-associated protein tau
MCI	mild cognitive impairment
MMSE	Mini-Mental State Examination
mPACC	modified Preclinical Alzheimer Cognitive Composite
MRI	magnetic resonance imaging
MS	mass spectrometry
MTBD	microtubule-binding domain
MT	Microtubule
NAD	non-Alzheimer’s disease condition
NFTs	neurofibrillary tangles
NTA	N-terminal tau
NTPD	N-terminal projection domain
PART	primary age-related tauopathy
PET	positron emission tomography
PP1	protein phosphatase 1
PP2A	protein phosphatase 2A
PP5	protein phosphatase 5
PRR	proline-rich region
PSEN1/2	presenilin-1/2
p-tau	phosphorylated tau
p-tau217	tau phosphorylated at threonine-217
p-tau181	tau phosphorylated at threonine-181
p-tau231	tau phosphorylated at threonine-231
PTEN	phosphatase and tensin homolog deleted on chromosome 10
SPECT	single-photon emission computed tomography
SUVR	standardized uptake value ratio
t-tau	total tau
TBI	traumatic brain injury
